# 1-Dichloro­acetyl-*r*-2,*c*-6-bis­(4-methoxy­phen­yl)-*c*-3,*t*-3-dimethyl­piperidin-4-one

**DOI:** 10.1107/S1600536808040063

**Published:** 2008-12-03

**Authors:** M. Thenmozhi, S. Ponnuswamy, V. Mohanraj, R. Vijayalakshmi, M. N. Ponnuswamy

**Affiliations:** a Centre of Advanced Study in Crystallography and Biophysics, University of Madras, Guindy Campus, Chennai 600 025, India; b Department of Chemistry, Government Arts College (Autonomous), Coimbatore 641 018, India; c Department of Chemistry, Queen Mary’s College (Autonomous), Chennai 600 004, India

## Abstract

In the title compound, C_23_H_25_Cl_2_NO_4_, the piperidine ring adopts a distorted boat conformation. Inversion-related mol­ecules are linked into centrosymmetric *R*_2_^2^(16) dimers by paired C—H⋯O hydrogen bonds, and the dimers are connected *via* C—H⋯O hydrogen bonds into a chain running along [101].

## Related literature

For general background, see: Eller *et al.*(2002 ▶); Ribeiro da Silva *et al.* (2007 ▶). For hybridization, see: Beddoes *et al.* (1986 ▶) For hydrogen-bond motifs, see: Bernstein *et al.* (1995 ▶). For ring conformational analysis, see: Cremer & Pople (1975 ▶); Nardelli (1983 ▶).
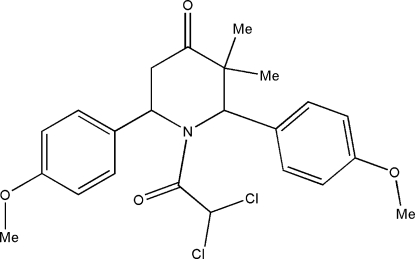


## Experimental

### 

#### Crystal data

C_23_H_25_Cl_2_NO_4_
*M* *_r_* = 450.34
Monoclinic, 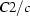

*a* = 23.6295 (9) Å
*b* = 10.3999 (4) Å
*c* = 19.2617 (9) Å
β = 107.734 (1)°
*V* = 4508.5 (3) Å^3^
*Z* = 8
Mo *K*α radiation
μ = 0.32 mm^−1^
*T* = 293 (2) K
0.30 × 0.25 × 0.20 mm

#### Data collection

Bruker Kappa APEXII area-detector diffractometer
Absorption correction: multi-scan (*SADABS*; Sheldrick, 2001 ▶) *T*_min_ = 0.911, *T*_max_ = 0.939
29354 measured reflections
6905 independent reflections
4138 reflections with *I* > 2σ(*I*)
*R*_int_ = 0.031

#### Refinement

*R*[*F* ^2^ > 2σ(*F* ^2^)] = 0.056
*wR*(*F* ^2^) = 0.184
*S* = 1.00
6905 reflections
275 parameters
H-atom parameters constrained
Δρ_max_ = 0.60 e Å^−3^
Δρ_min_ = −0.63 e Å^−3^

### 

Data collection: *APEX2* (Bruker, 2004 ▶); cell refinement: *APEX2*; data reduction: *SAINT* (Bruker, 2004 ▶); program(s) used to solve structure: *SHELXS97* (Sheldrick, 2008 ▶); program(s) used to refine structure: *SHELXS97* (Sheldrick, 2008 ▶); molecular graphics: *ORTEP-3* (Farrugia, 1997 ▶); software used to prepare material for publication: *SHELXL97* and *PLATON* (Spek, 2003 ▶).

## Supplementary Material

Crystal structure: contains datablocks global, I. DOI: 10.1107/S1600536808040063/ci2726sup1.cif

Structure factors: contains datablocks I. DOI: 10.1107/S1600536808040063/ci2726Isup2.hkl

Additional supplementary materials:  crystallographic information; 3D view; checkCIF report

## Figures and Tables

**Table 1:** Hydrogen-bond geometry (Å, °)

*D*—H⋯*A*	*D*—H	H⋯*A*	*D*⋯*A*	*D*—H⋯*A*
C6—H6⋯O1^i^	0.98	2.30	3.216 (2)	155
C13—H13⋯O3^ii^	0.93	2.58	3.453 (3)	155

Symmetry codes: (i) 
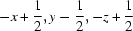
; (ii) 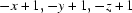.

**Fig. 1.**
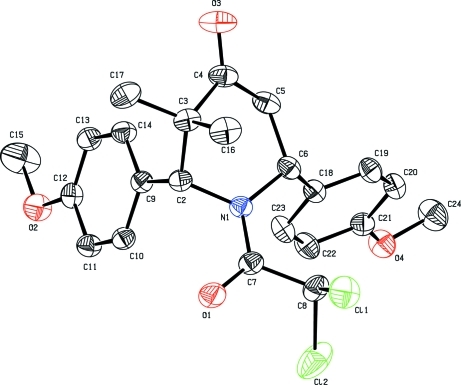
The molecular structure of the title compound, showing 50% probability displacement ellipsoids.

**Fig. 2.**
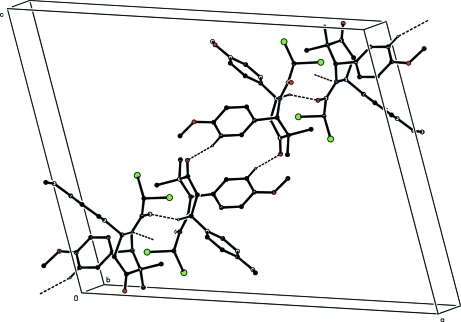
The crystal packing of the molecules viewed down b axis. Hydrogen bonds are shown as dashed lines.

## supplementary crystallographic information

### Comment

The piperidine compounds are used in chemical industry for the synthesis of pharmacological drugs, either as reactants, solvents or being units of molecular chemical structure of the final compounds. These compounds have significant biological importance with more environmental impact (Ribeiro da Silva *et al.*, 2007). A significant industrial application of piperidine is for the production of dipiperidinyl dithiuram tetrasulfide, which is used as a rubber vulcanization accelerator (Eller *et al.*, 2002).

The piperidine ring adopts a distorted boat conformation, with puckering parameters (Cremer & Pople, 1975) q_2_ = 0.606 (2) Å, q_3_ = 0.127 (2) Å, and φ = 77.5 (2)°, and asymmetry parameter Δ_s_(C2) = 18.26 (17)° (Nardelli, 1983). The torsion angles C13—C12—O2—C15 [2.6 (3)°] and C20—C21—O4—C24 [-11.7 (3)°] indicate that the methoxy groups are almost coplanar with the attached rings (Fig.1). The sum of the bond angles around atom N1 (359.4°) of the piperidine ring is in accordance with *sp*^2^ hybridization (Beddoes *et al.*, 1986). The best plane through the piperidine ring (N1/C3/C4/C6) and methoxyphenyl ring (C9—C14) are orthogonal to one another, with a dihedral angle of 87.18 (7)°, whereas, the other methoxyphenyl ring (C18—C23) is oriented at an angle of 54.47 (7)°.

The molecules at positions (*x*, *y*, *z*) and (1 - *x*, 1 - *y*, 1 - *z*) are linked through a pair of C13—H13···O3 hydrogen bonds forming a cyclic centrosymmetric *R*_2_^2^(16) dimer (Bernstein *et al.*, 1995). The dimers are linked by intermolecular C6—H6···O1 hydrogen bonds (Table 1) into a chain running along the [101] (Fig.2).

### Experimental

To the solution of r-2,c-6-bis(4-methoxyphenyl)-c-3,t-3-dimethylpiperidin-4-one (2 g) in benzene (25 ml), triethylamine (2.0 ml) and dichloroacetyl chloride (1.40 ml) were added and allowed to reflux on a water bath for 5 h. The course of the reaction was monitored by TLC. The solution was concentrated and the resulting mass was crystallized from ethanol.

### Refinement

H atoms were positioned geometrically (C—H = 0.93–0.98 Å) and allowed to ride on their parent atoms, with *U*_iso_(H) = 1.2–1.5(methyl) *U*_eq_(C).

### Crystal data

**Table:**

C_23_H_25_Cl_2_NO_4_	*F*(000) = 1888
*M**_r_* = 450.34	*D*_x_ = 1.327 Mg m^−^^3^
Monoclinic, *C*2/*c*	Melting point = 377–379 K
Hall symbol: -C 2yc	Mo *K*α radiation, λ = 0.71073 Å
*a* = 23.6295 (9) Å	Cell parameters from 6905 reflections
*b* = 10.3999 (4) Å	θ = 2.2–30.6°
*c* = 19.2617 (9) Å	µ = 0.32 mm^−^^1^
β = 107.734 (1)°	*T* = 293 K
*V* = 4508.5 (3) Å^3^	Block, colourless
*Z* = 8	0.30 × 0.25 × 0.20 mm

### Data collection

**Table:**

Bruker Kappa APEXII area-detector diffractometer	6905 independent reflections
Radiation source: fine-focus sealed tube	4138 reflections with *I* > 2σ(*I*)
graphite	*R*_int_ = 0.031
ω and φ scans	θ_max_ = 30.6°, θ_min_ = 2.2°
Absorption correction: multi-scan (*SADABS*; Sheldrick, 2001)	*h* = −32→33
*T*_min_ = 0.911, *T*_max_ = 0.939	*k* = −14→14
29354 measured reflections	*l* = −27→27

### Refinement

**Table:**

Refinement on *F*^2^	Primary atom site location: structure-invariant direct methods
Least-squares matrix: full	Secondary atom site location: difference Fourier map
*R*[*F*^2^ > 2σ(*F*^2^)] = 0.056	Hydrogen site location: inferred from neighbouring sites
*wR*(*F*^2^) = 0.184	H-atom parameters constrained
*S* = 1.00	*w* = 1/[σ^2^(*F*_o_^2^) + (0.0869*P*)^2^ + 2.9239*P*] where *P* = (*F*_o_^2^ + 2*F*_c_^2^)/3
6905 reflections	(Δ/σ)_max_ = 0.001
275 parameters	Δρ_max_ = 0.60 e Å^−^^3^
0 restraints	Δρ_min_ = −0.63 e Å^−^^3^

### Special details

**Table:**

Geometry. All e.s.d.'s (except the e.s.d. in the dihedral angle between two l.s. planes) are estimated using the full covariance matrix. The cell e.s.d.'s are taken into account individually in the estimation of e.s.d.'s in distances, angles and torsion angles; correlations between e.s.d.'s in cell parameters are only used when they are defined by crystal symmetry. An approximate (isotropic) treatment of cell e.s.d.'s is used for estimating e.s.d.'s involving l.s. planes.
Refinement. Refinement of *F*^2^ against ALL reflections. The weighted *R*-factor *wR* and goodness of fit *S* are based on *F*^2^, conventional *R*-factors *R* are based on *F*, with *F* set to zero for negative *F*^2^. The threshold expression of *F*^2^ > σ(*F*^2^) is used only for calculating *R*-factors(gt) *etc*. and is not relevant to the choice of reflections for refinement. *R*-factors based on *F*^2^ are statistically about twice as large as those based on *F*, and *R*- factors based on ALL data will be even larger.

### Fractional atomic coordinates and isotropic or equivalent isotropic displacement parameters (Å^2^)

**Table:**

	*x*	*y*	*z*	*U*_iso_*/*U*_eq_	
Cl1	0.18029 (3)	0.52254 (7)	0.14172 (4)	0.0790 (2)	
Cl2	0.26274 (4)	0.62339 (11)	0.07261 (4)	0.1127 (4)	
O1	0.26407 (7)	0.74756 (16)	0.21261 (9)	0.0635 (4)	
O2	0.56012 (6)	0.89254 (16)	0.36906 (9)	0.0604 (4)	
O3	0.37036 (9)	0.4204 (2)	0.48165 (10)	0.0798 (6)	
O4	0.48349 (7)	0.30777 (15)	0.10880 (9)	0.0592 (4)	
N1	0.31916 (6)	0.58476 (15)	0.27717 (8)	0.0402 (3)	
C2	0.33601 (8)	0.66580 (19)	0.34377 (10)	0.0407 (4)	
H2	0.3092	0.7400	0.3325	0.049*	
C3	0.32172 (8)	0.5947 (2)	0.40675 (11)	0.0481 (5)	
C4	0.35641 (9)	0.4707 (2)	0.42265 (12)	0.0530 (5)	
C5	0.37393 (9)	0.4121 (2)	0.36076 (12)	0.0521 (5)	
H5A	0.3721	0.3194	0.3651	0.063*	
H5B	0.4151	0.4343	0.3676	0.063*	
C6	0.33818 (8)	0.44832 (19)	0.28249 (11)	0.0435 (4)	
H6	0.3023	0.3950	0.2685	0.052*	
C7	0.28061 (8)	0.6362 (2)	0.21725 (11)	0.0460 (4)	
C8	0.25568 (9)	0.5486 (2)	0.15090 (12)	0.0550 (5)	
H8	0.2771	0.4665	0.1587	0.066*	
C9	0.39837 (8)	0.72041 (18)	0.35651 (10)	0.0402 (4)	
C10	0.40476 (8)	0.81147 (19)	0.30699 (10)	0.0431 (4)	
H10	0.3716	0.8357	0.2689	0.052*	
C11	0.45865 (9)	0.8669 (2)	0.31249 (11)	0.0476 (5)	
H11	0.4616	0.9282	0.2786	0.057*	
C12	0.50864 (8)	0.83169 (19)	0.36834 (11)	0.0453 (4)	
C13	0.50374 (8)	0.7419 (2)	0.41826 (11)	0.0475 (5)	
H13	0.5371	0.7176	0.4560	0.057*	
C14	0.44863 (8)	0.6872 (2)	0.41224 (11)	0.0474 (5)	
H14	0.4456	0.6268	0.4466	0.057*	
C15	0.61272 (11)	0.8573 (4)	0.4232 (2)	0.1002 (11)	
H15A	0.6096	0.8793	0.4703	0.150*	
H15B	0.6457	0.9022	0.4154	0.150*	
H15C	0.6188	0.7663	0.4210	0.150*	
C16	0.25568 (9)	0.5548 (3)	0.38209 (14)	0.0630 (6)	
H16A	0.2487	0.4941	0.3428	0.095*	
H16B	0.2313	0.6293	0.3659	0.095*	
H16C	0.2459	0.5160	0.4222	0.095*	
C17	0.33296 (11)	0.6809 (3)	0.47360 (12)	0.0646 (6)	
H17A	0.3258	0.6332	0.5128	0.097*	
H17B	0.3067	0.7536	0.4621	0.097*	
H17C	0.3734	0.7101	0.4881	0.097*	
C18	0.37492 (8)	0.41315 (18)	0.23272 (11)	0.0416 (4)	
C19	0.37935 (9)	0.2855 (2)	0.21607 (12)	0.0498 (5)	
H19	0.3576	0.2247	0.2326	0.060*	
C20	0.41538 (9)	0.2454 (2)	0.17531 (12)	0.0507 (5)	
H20	0.4180	0.1587	0.1650	0.061*	
C21	0.44721 (8)	0.3353 (2)	0.15026 (11)	0.0458 (4)	
C22	0.44278 (9)	0.4630 (2)	0.16603 (13)	0.0533 (5)	
H22	0.4640	0.5240	0.1488	0.064*	
C23	0.40738 (9)	0.5018 (2)	0.20705 (13)	0.0508 (5)	
H23	0.4052	0.5885	0.2177	0.061*	
C24	0.49748 (14)	0.1779 (2)	0.10196 (17)	0.0750 (7)	
H24A	0.5128	0.1402	0.1495	0.113*	
H24B	0.5269	0.1721	0.0770	0.113*	
H24C	0.4623	0.1326	0.0747	0.113*	

### Atomic displacement parameters (Å^2^)

**Table:**

	*U*^11^	*U*^22^	*U*^33^	*U*^12^	*U*^13^	*U*^23^
Cl1	0.0512 (3)	0.0880 (5)	0.0926 (5)	−0.0197 (3)	0.0140 (3)	0.0036 (4)
Cl2	0.1188 (7)	0.1699 (10)	0.0577 (4)	−0.0632 (6)	0.0394 (4)	−0.0210 (5)
O1	0.0641 (9)	0.0589 (10)	0.0558 (9)	0.0225 (8)	0.0008 (7)	0.0010 (7)
O2	0.0427 (7)	0.0669 (10)	0.0689 (10)	−0.0084 (7)	0.0130 (7)	0.0073 (8)
O3	0.0847 (13)	0.0963 (14)	0.0611 (11)	0.0211 (11)	0.0265 (9)	0.0364 (10)
O4	0.0632 (9)	0.0547 (9)	0.0686 (10)	0.0045 (7)	0.0332 (8)	−0.0007 (8)
N1	0.0348 (7)	0.0415 (8)	0.0421 (8)	0.0034 (6)	0.0087 (6)	0.0038 (7)
C2	0.0355 (8)	0.0449 (10)	0.0413 (9)	0.0042 (7)	0.0110 (7)	0.0027 (8)
C3	0.0389 (9)	0.0617 (13)	0.0448 (10)	0.0023 (8)	0.0146 (8)	0.0073 (9)
C4	0.0407 (9)	0.0641 (14)	0.0543 (12)	0.0010 (9)	0.0148 (9)	0.0185 (10)
C5	0.0486 (10)	0.0480 (12)	0.0609 (13)	0.0096 (9)	0.0183 (9)	0.0164 (10)
C6	0.0369 (8)	0.0398 (10)	0.0541 (11)	−0.0008 (7)	0.0143 (8)	0.0053 (8)
C7	0.0381 (9)	0.0522 (12)	0.0449 (10)	0.0084 (8)	0.0084 (8)	0.0022 (9)
C8	0.0396 (9)	0.0657 (14)	0.0514 (12)	0.0050 (9)	0.0015 (8)	−0.0046 (10)
C9	0.0384 (8)	0.0412 (10)	0.0408 (9)	0.0021 (7)	0.0117 (7)	0.0000 (8)
C10	0.0394 (9)	0.0470 (11)	0.0412 (9)	0.0023 (8)	0.0097 (7)	0.0009 (8)
C11	0.0477 (10)	0.0495 (11)	0.0462 (11)	−0.0009 (8)	0.0151 (8)	0.0052 (9)
C12	0.0398 (9)	0.0460 (11)	0.0505 (11)	−0.0016 (8)	0.0145 (8)	−0.0059 (9)
C13	0.0386 (9)	0.0509 (12)	0.0479 (11)	0.0033 (8)	0.0057 (8)	0.0023 (9)
C14	0.0426 (9)	0.0511 (12)	0.0457 (10)	0.0006 (8)	0.0093 (8)	0.0080 (9)
C15	0.0412 (12)	0.117 (3)	0.126 (3)	−0.0143 (14)	0.0005 (14)	0.044 (2)
C16	0.0400 (10)	0.0862 (18)	0.0664 (14)	−0.0007 (10)	0.0214 (10)	0.0106 (13)
C17	0.0607 (13)	0.0883 (18)	0.0478 (12)	0.0039 (12)	0.0212 (10)	0.0006 (12)
C18	0.0352 (8)	0.0387 (10)	0.0502 (10)	0.0000 (7)	0.0119 (7)	0.0025 (8)
C19	0.0501 (10)	0.0419 (11)	0.0592 (12)	−0.0088 (8)	0.0193 (9)	0.0019 (9)
C20	0.0554 (11)	0.0383 (11)	0.0584 (12)	−0.0018 (9)	0.0172 (10)	−0.0034 (9)
C21	0.0419 (9)	0.0483 (11)	0.0463 (10)	0.0025 (8)	0.0122 (8)	0.0024 (9)
C22	0.0521 (11)	0.0441 (11)	0.0693 (14)	−0.0048 (9)	0.0268 (10)	0.0041 (10)
C23	0.0504 (11)	0.0363 (10)	0.0708 (14)	−0.0005 (8)	0.0258 (10)	0.0009 (9)
C24	0.0880 (18)	0.0611 (16)	0.0897 (19)	0.0135 (13)	0.0475 (16)	−0.0023 (14)

### Geometric parameters (Å, °)

**Table:**

Cl1—C8	1.757 (2)	C11—C12	1.383 (3)
Cl2—C8	1.749 (2)	C11—H11	0.93
O1—C7	1.217 (2)	C12—C13	1.370 (3)
O2—C12	1.368 (2)	C13—C14	1.393 (3)
O2—C15	1.406 (3)	C13—H13	0.93
O3—C4	1.202 (3)	C14—H14	0.93
O4—C21	1.368 (2)	C15—H15A	0.96
O4—C24	1.406 (3)	C15—H15B	0.96
N1—C7	1.344 (2)	C15—H15C	0.96
N1—C6	1.483 (2)	C16—H16A	0.96
N1—C2	1.484 (2)	C16—H16B	0.96
C2—C9	1.528 (2)	C16—H16C	0.96
C2—C3	1.544 (3)	C17—H17A	0.96
C2—H2	0.98	C17—H17B	0.96
C3—C4	1.508 (3)	C17—H17C	0.96
C3—C17	1.524 (3)	C18—C19	1.377 (3)
C3—C16	1.543 (3)	C18—C23	1.383 (3)
C4—C5	1.505 (3)	C19—C20	1.387 (3)
C5—C6	1.534 (3)	C19—H19	0.93
C5—H5A	0.97	C20—C21	1.376 (3)
C5—H5B	0.97	C20—H20	0.93
C6—C18	1.521 (3)	C21—C22	1.373 (3)
C6—H6	0.98	C22—C23	1.374 (3)
C7—C8	1.533 (3)	C22—H22	0.93
C8—H8	0.98	C23—H23	0.93
C9—C14	1.380 (3)	C24—H24A	0.96
C9—C10	1.385 (3)	C24—H24B	0.96
C10—C11	1.372 (3)	C24—H24C	0.96
C10—H10	0.93		
			
C12—O2—C15	117.99 (19)	O2—C12—C11	115.57 (18)
C21—O4—C24	117.58 (18)	C13—C12—C11	119.45 (18)
C7—N1—C6	123.51 (16)	C12—C13—C14	119.78 (18)
C7—N1—C2	116.70 (15)	C12—C13—H13	120.1
C6—N1—C2	119.19 (15)	C14—C13—H13	120.1
N1—C2—C9	109.97 (14)	C9—C14—C13	121.55 (19)
N1—C2—C3	109.99 (16)	C9—C14—H14	119.2
C9—C2—C3	118.89 (16)	C13—C14—H14	119.2
N1—C2—H2	105.7	O2—C15—H15A	109.5
C9—C2—H2	105.7	O2—C15—H15B	109.5
C3—C2—H2	105.7	H15A—C15—H15B	109.5
C4—C3—C17	112.76 (18)	O2—C15—H15C	109.5
C4—C3—C16	105.59 (19)	H15A—C15—H15C	109.5
C17—C3—C16	108.54 (17)	H15B—C15—H15C	109.5
C4—C3—C2	109.49 (15)	C3—C16—H16A	109.5
C17—C3—C2	111.01 (18)	C3—C16—H16B	109.5
C16—C3—C2	109.25 (17)	H16A—C16—H16B	109.5
O3—C4—C5	120.7 (2)	C3—C16—H16C	109.5
O3—C4—C3	122.7 (2)	H16A—C16—H16C	109.5
C5—C4—C3	116.68 (17)	H16B—C16—H16C	109.5
C4—C5—C6	118.50 (17)	C3—C17—H17A	109.5
C4—C5—H5A	107.7	C3—C17—H17B	109.5
C6—C5—H5A	107.7	H17A—C17—H17B	109.5
C4—C5—H5B	107.7	C3—C17—H17C	109.5
C6—C5—H5B	107.7	H17A—C17—H17C	109.5
H5A—C5—H5B	107.1	H17B—C17—H17C	109.5
N1—C6—C18	113.94 (15)	C19—C18—C23	117.99 (18)
N1—C6—C5	111.57 (16)	C19—C18—C6	118.50 (17)
C18—C6—C5	108.14 (15)	C23—C18—C6	123.37 (18)
N1—C6—H6	107.6	C18—C19—C20	121.69 (18)
C18—C6—H6	107.6	C18—C19—H19	119.2
C5—C6—H6	107.6	C20—C19—H19	119.2
O1—C7—N1	124.06 (19)	C21—C20—C19	119.30 (19)
O1—C7—C8	118.08 (18)	C21—C20—H20	120.3
N1—C7—C8	117.85 (18)	C19—C20—H20	120.3
C7—C8—Cl2	109.96 (16)	O4—C21—C22	115.80 (18)
C7—C8—Cl1	107.21 (15)	O4—C21—C20	124.66 (19)
Cl2—C8—Cl1	110.00 (12)	C22—C21—C20	119.54 (18)
C7—C8—H8	109.9	C21—C22—C23	120.75 (19)
Cl2—C8—H8	109.9	C21—C22—H22	119.6
Cl1—C8—H8	109.9	C23—C22—H22	119.6
C14—C9—C10	117.29 (17)	C22—C23—C18	120.72 (19)
C14—C9—C2	126.23 (17)	C22—C23—H23	119.6
C10—C9—C2	116.48 (16)	C18—C23—H23	119.6
C11—C10—C9	121.84 (18)	O4—C24—H24A	109.5
C11—C10—H10	119.1	O4—C24—H24B	109.5
C9—C10—H10	119.1	H24A—C24—H24B	109.5
C10—C11—C12	120.10 (19)	O4—C24—H24C	109.5
C10—C11—H11	120.0	H24A—C24—H24C	109.5
C12—C11—H11	120.0	H24B—C24—H24C	109.5
O2—C12—C13	124.98 (18)		
			
C7—N1—C2—C9	−104.12 (18)	N1—C2—C9—C14	−110.8 (2)
C6—N1—C2—C9	84.47 (19)	C3—C2—C9—C14	17.3 (3)
C7—N1—C2—C3	123.10 (17)	N1—C2—C9—C10	68.9 (2)
C6—N1—C2—C3	−48.31 (19)	C3—C2—C9—C10	−163.05 (18)
N1—C2—C3—C4	60.4 (2)	C14—C9—C10—C11	0.1 (3)
C9—C2—C3—C4	−67.6 (2)	C2—C9—C10—C11	−179.62 (18)
N1—C2—C3—C17	−174.47 (15)	C9—C10—C11—C12	0.4 (3)
C9—C2—C3—C17	57.5 (2)	C15—O2—C12—C13	2.6 (3)
N1—C2—C3—C16	−54.8 (2)	C15—O2—C12—C11	−177.9 (3)
C9—C2—C3—C16	177.18 (18)	C10—C11—C12—O2	−179.95 (18)
C17—C3—C4—O3	29.1 (3)	C10—C11—C12—C13	−0.5 (3)
C16—C3—C4—O3	−89.2 (3)	O2—C12—C13—C14	179.43 (19)
C2—C3—C4—O3	153.3 (2)	C11—C12—C13—C14	0.0 (3)
C17—C3—C4—C5	−150.11 (19)	C10—C9—C14—C13	−0.6 (3)
C16—C3—C4—C5	91.5 (2)	C2—C9—C14—C13	179.11 (18)
C2—C3—C4—C5	−26.0 (2)	C12—C13—C14—C9	0.5 (3)
O3—C4—C5—C6	158.4 (2)	N1—C6—C18—C19	−160.22 (17)
C3—C4—C5—C6	−22.3 (3)	C5—C6—C18—C19	75.1 (2)
C7—N1—C6—C18	66.7 (2)	N1—C6—C18—C23	24.2 (3)
C2—N1—C6—C18	−122.54 (17)	C5—C6—C18—C23	−100.5 (2)
C7—N1—C6—C5	−170.53 (17)	C23—C18—C19—C20	0.4 (3)
C2—N1—C6—C5	0.3 (2)	C6—C18—C19—C20	−175.39 (19)
C4—C5—C6—N1	36.3 (2)	C18—C19—C20—C21	−0.5 (3)
C4—C5—C6—C18	162.32 (18)	C24—O4—C21—C22	169.2 (2)
C6—N1—C7—O1	178.95 (18)	C24—O4—C21—C20	−11.7 (3)
C2—N1—C7—O1	7.9 (3)	C19—C20—C21—O4	−179.0 (2)
C6—N1—C7—C8	−0.6 (3)	C19—C20—C21—C22	0.0 (3)
C2—N1—C7—C8	−171.63 (16)	O4—C21—C22—C23	179.7 (2)
O1—C7—C8—Cl2	49.9 (2)	C20—C21—C22—C23	0.6 (3)
N1—C7—C8—Cl2	−130.51 (17)	C21—C22—C23—C18	−0.7 (4)
O1—C7—C8—Cl1	−69.7 (2)	C19—C18—C23—C22	0.2 (3)
N1—C7—C8—Cl1	109.92 (18)	C6—C18—C23—C22	175.8 (2)

### Hydrogen-bond geometry (Å, °)

**Table:**

*D*—H···*A*	*D*—H	H···*A*	*D*···*A*	*D*—H···*A*
C6—H6···O1^i^	0.98	2.30	3.216 (2)	155
C13—H13···O3^ii^	0.93	2.58	3.453 (3)	155

Symmetry codes: (i) −*x*+1/2, *y*−1/2, −*z*+1/2; (ii) −*x*+1, −*y*+1, −*z*+1.
